# Decrease in Sperm Count After Bariatric Surgery: Case Reports

**DOI:** 10.7759/cureus.20388

**Published:** 2021-12-13

**Authors:** Aneela Razzaq, Faiza H Soomro, Ghulam Siddiq, Samina Khizar, Murad Ali Khan

**Affiliations:** 1 Surgery, Wexford General Hospital, Wexford, IRL; 2 General Surgery, Shifa International Hospital, Islamabad, PAK; 3 General Surgery, The Dudley Group National Health Service Foundation Trust, Dudley, GBR

**Keywords:** bariatric surgery, semen analysis, infertility, weight loss, azospermia

## Abstract

Morbid obesity is associated with a large number of complications, including infertility; weight loss can help to improve fertility and increase the number of sperm in males. However, two of our patients developed azoospermia after bariatric surgery for weight reduction.

A 30-year-old male presented to the outpatient department (OPD) with a BMI of 81.2 kg/m^2^ (258 kg) with no known co-morbidities. The patient had a normal sex life and one child. After bariatric surgery, he noticed a change in the consistency of his semen and azoospermia. In the second case, a 48-year-old man presented to the OPD with a BMI of 52 kg/m^2^ (189 kg) with no known co-morbidities. He had three children. He underwent bariatric surgery for weight loss and, after one year, he developed azoospermia.

Bariatric surgery is a lifesaving procedure for morbidly obese patients and helps in restoring normal daily activities. This weight reduction surgery helps in decreasing blood pressure, increasing glycemic control and improving sexual activity. However, bariatric surgery may be followed by a further decline in semen parameters, resulting in azoospermia and severe oligoasthenoteratozoospermia. This is caused by the combined effects of two different processes: 1) the subduing of the negative effects of obesity, and 2) a deficiency of nutrients along with the release of some harmful substances. Bariatric surgery patients should be informed about the risk of complications and about the possibility for cryopreservation of sperm.

In rare cases, bariatric surgery can result in a decrease in sperm count and infertility in males.

## Introduction

Obesity is an increasingly serious public health problem worldwide and is associated with multiple health problems, including increased risk for cardiovascular disorders, diabetes and reduced life expectancy [1]. Obesity is also reported to be associated with reproductive abnormalities, including hypogonadism, impaired sperm quality [2] and diminished sexual quality of life [3].

The reproductive function, which is controlled by hormones, is negatively affected by obesity. For example, adipocytes, which are fat cells, increase the production of leptin in pathologically obese women, causing changes in the menstrual cycle and decreasing fertility in these women. The chances of poly cystic ovarian syndrome are increased in obese women as obesity causes anovulation. Obesity in males impacts their fertility by decreasing the total number of sperms and increasing the number of slow-swimming sperms. Obesity is also involved in altering the levels of hormones, including testosterone, follicle-stimulating hormone and sex hormone-binding globulin in both genders, which causes infertility [4,5].

Although behavioral and pharmacologic weight-loss therapies are well established, bariatric surgery has proven to be an effective treatment strategy for obesity and is associated with an improved quality of life and comorbidities. The most commonly conducted bariatric surgical procedures are Roux-en-Y gastric bypass, sleeve gastrectomy and laparoscopic adjustable gastric band [6]. 

In laparoscopic adjustable gastric band surgery, an expandable elastic bag is attached and tightened around the stomach so that a person feels full even after eating a small amount of food. In sleeve gastrectomy, part of the stomach is removed to decrease its volume and, thus, limit the volume of consumed food. In gastric bypass surgery, both malabsorptive and restrictive methods are used as not only is the size of the stomach decreased, but the small intestine is also re-shaped to bypass a major portion of the stomach and, thus, restrict the amount of calories absorbed. A report that compares bariatric surgery and natural gradual weight loss showed that, in women, rapid weight loss as a result of bariatric surgery is more effective in increasing fertility [7].

## Case presentation

Case 1

A 30-year-old male presented to our surgical outpatient department after being referred from the medicine department with a complaint of morbid obesity. The patient had obstructive sleep apnea, impaired glucose tolerance and hypertension that was difficult to control on antihypertensive medication. The patient had a BMI of 81.2 kg/m^2^ (258 kg) and has own hotel business. Initially, pharmacological treatment along with physical activity was started by the medicine unit, but the patient’s weight and symptoms were refractory to conservative management. He was investigated, and his hormonal profile was normal. The patient had been married for four years and had one child and a normal reproductive life. Bariatric surgery was planned in a multidisciplinary meeting that included the surgery, anaesthesia, medicine and cardiology departments. The patient and his family were informed about the benefits and risks of the treatment, and the patient underwent laparoscopic sleeve gastrectomy in 2014, which was successful; the immediate post-operative period was unremarkable, and the patient was discharged home with multi-vitamins and in order to maintain a proper calories dietitian was also involved. The patient had regular follow-ups, which showed no active issues. The patient was losing weight, and his symptoms of obstructive sleep apnea improved. He started his routine life activities and had a good sleep pattern. He was weaned from antihypertensive medication, and glucose control was optimal without any medication. At the patient’s last visit in March 2018, he had achieved a BMI of 46.4 kg/m^2^ (147 kg). The only abnormality was that the patient and his wife has been unable to conceive in the four years following his surgery; therefore, an initial workup, including semen analysis, was done. He also complained about a change in the consistency of his semen. Semen analysis showed nil sperm count, and the patient was diagnosed with azoospermia by repeating sample after seven days of sexual abstinence and consultation with a urologist.

Case 2

The second case was a 48-year-old man who presented to our department with morbid obesity and restricted life activities due to excessive weight gain. The patient’s appetite was normal. The patient had a BMI of 52 kg/m^2^ (189 kg) with no known co-morbidities. The patient had no addictions and was not taking any medicines regularly. The patient had been married for nine years and had three children with a normal reproductive life and was a lecturer in a university. The patient had a history of follow-up with a nutritionist and a physical trainer, but his weight was not decreasing. Bariatric surgery was decided upon in a multidisciplinary meeting, and the family agreed to the treatment plan. The patient underwent laparoscopic sleeve gastrectomy in 2016. He experienced no immediate complications and he was discharged with multi-vitamins and regular dietitian consultation to meet his caloric requirements. He also attended regular follow-ups. The patient was losing weight successfully and starting daily routine life activities. One year following his bariatric procedure, he had lost 68 kg and achieved a BMI of 41.2 kg/m^2^; he had also married his second wife. They had not been able to conceive, and he was investigated for infertility, which found no hormonal or reproductive issues. He then underwent semen analysis and repeating sample after 7 days of sexual abstinence it showed azoospermia and a urologist consultation was done (Table 1).

**Table 1:** Semen Analysis

PARAMETERS	CASE 1	CASE 2
Volume	4 ml	2 ml
Colour	Grey white	Opaque white
pH	7.7	8.0
Sperm count	Nil	Nil
Active motile	Nil	Nil
Sluggish motile	Nil	Nil
Morphology	Nil	Nil
Opinion	Azoospermia	Azoospermia

## Discussion

The link between reduced sperm count and weight loss by bariatric surgery is multifaceted, and various theories have been proposed, both pathological and physiological. (1) First, regular gonadotrophin release is affected by rapid weight loss as sometimes it stimulates an undernutrition status. (2) Bariatric surgeries, including sleeve gastrectomy and gastric bypass surgery, can lead to various types of nutritional deficiencies, including calcium, vitamins B, B6, and B12 and iron, which are all involved in spermatogenesis. To compensate for these nutritional deficiencies, vitamins and mineral supplements are recommended after bariatric surgery. It is also hypothesised that multiple lipid-soluble toxic substances are released after bariatric surgery, which, in addition to rapid weight loss, affect sperm formation [7,8].

Most previous studies have shown beneficial effects of bariatric surgery on infertility and increased sperm count [9-12]. Only a few case series have reported decreased sperm count and azoospermia after bariatric surgery [13]. One study included 42 men with obesity (body mass index over 40 kg/m²). As a comparison, it also included 32 men without obesity (BMI under 35 kg/m²) who had fathered at least two children. All of the men underwent blood tests for hormone levels and provided semen samples. Eighteen men with obesity had bariatric surgery. They were followed up after six months and compared to 14 men with obesity who did not have surgery. The obese men had significantly higher estrogen (estradiol 33.3 vs. 22.0 pg/ml) and lower total testosterone (272.5 vs. 413 ng/dl) compared to the non-obese men. The patients with obesity had a significantly lower sperm count as compared to normal-weight men (82.5 vs. 205.2 million). After bariatric surgery, the patients’ body weight significantly decreased, and they had lower rates of high blood pressure and diabetes. In addition, their total testosterone levels significantly increased following surgery (604 vs. 294.5 ng/dl). Sexual desire also improved after surgery. However, their sperm count decreased dramatically following surgery (122.8 million to 17.0 million). Two patients (11%) no longer produced sperm following surgery (azoospermia) [13]. In another case series, six patients were reported to experience azoospermia, presenting secondary infertility after Roux-en-Y gastric bypass interventions [14]. Another series of three cases reported a worsening of semen parameters after bariatric surgery, including oligoasthenoteratozoospermia [15].

In another case series, 46 adult men who underwent gastric bypass (n = 20) or sleeve gastrectomy (n = 26) were included. Total sperm count tended to be lower at six months following surgery and showed a significant decrease at 12 months in both surgery groups, and there was a decrease of 54.6 million (96.8 to 42.2 million; P = 0.0021). Total sperm count at 12 months was 41.4 million less than the baseline sperm count (P = 0.0391) after gastric bypass and 91.1 million (P = 0.0080) after sleeve gastrectomy. There was no case of azoospermia [16].

## Conclusions

Patients must be informed about the possible reproductive complications of bariatric surgery, even if few patients experience these complications. Future research should be conducted to further evaluate the ways in which both natural gradual weight loss by diet modification and bariatric surgery affect fertility and sperm parameters.
